# New screening system using Twist1 promoter activity identifies dihydrorotenone as a potent drug targeting cancer-associated fibroblasts

**DOI:** 10.1038/s41598-020-63996-4

**Published:** 2020-04-27

**Authors:** Eunmyong Lee, So-Young Yeo, Keun-Woo Lee, Jin A. Lee, Kyeong Kyu Kim, Seok-Hyung Kim

**Affiliations:** 1 Samsung Biomedical Research Institute, Sungkyunkwan University School of Medicine, Seoul, Korea; 2 Department of Health Science and Technology, Samsung Advanced Institute for Health Science and Technology, Sungkyunkwan University, Seoul, Republic of Korea; 3 Department of Molecular Cell Biology, Sungkyunkwan University School of Medicine, Suwon, Korea; 4 Department of Pathology, Samsung Medical Center, Sungkyunkwan University School of Medicine, Seoul, Korea

**Keywords:** Cancer microenvironment, High-throughput screening

## Abstract

Cancer-associated fibroblasts (CAFs) are the most abundant stromal cells in tumor microenvironments. These cells strongly support tumor progression and are considered to be potent therapeutic targets. Therefore, drugs targeting CAFs have been developed, but most of them have failed in clinical trials. The discovery of additional drugs to inactivate or eliminate CAFs is thus essential. In this study, we developed a high-throughput screening system to find anti-CAF drugs using reporter cells that express Twist1 promoter-GFP. This screening system uses the activity of the Twist1 promoter as an indicator of CAF activation because Twist1 is known to be a central player in CAF activation. Using this screening system, we found that dihydrorotenone (DHR), an inhibitor of electron transfer chain complex 1 in mitochondria, can effectively deactivate CAFs. DHR-treated CAFs exhibited reduced expression of CAF-enriched markers, decreased capability of collagen gel contraction, and impaired ability to engage in tumor-promoting activities, such as facilitating the proliferation and colonization of cancer cells. Furthermore, conditioned media from DHR-treated CAFs attenuated tumor progression in mice grafted with MNK28 cells. In conclusion, DHR can be considered as a candidate drug targeting CAFs.

## Introduction

Fibroblasts play a key role in maintaining tissue homeostasis by contributing to tissue repair during wound healing. In this process, quiescent fibroblasts become activated to support tissue repair, and then they return to their initial state after tissue recovery^1,2^. During the early stages of cancer development, fibroblasts restrict tumor progression and maintain tissue homeostasis^3^. To overcome that limit, cancer cells transform normal fibroblasts (NFs) into cancer-associated fibroblasts (CAFs), which support tumor progression^2^.

CAFs, the major component of tumor stroma, interact with tumor cells and facilitate tumor progression and metastasis^1^. A large proportion of CAFs are activated fibroblasts that express fibroblast activation markers such as FSP1, FAP, PDGFR, and α-SMA^1,4^. They acquire proliferation capacity and synthesize abundant paracrine factors and extracellular matrix (ECM) components^1^. A series of pro-inflammatory gene sets active in CAFs have been known to recruit macrophages, resulting in an immunosuppressive environment and tumor promotion^5,6^. In addition, CAFs directly activate the survival pathway in tumor cells via paracrine factors, and CAF-mediated ECM remodeling facilitates the dissemination of tumor cells^1^.

Despite growing evidence for the central role of CAFs in promoting tumor progression, very little is known about the regulating factors responsible for the differentiation of NFs into CAFs. Recent studies have indicated that Twist1 is a key regulator of CAF activation^7–9^. As a transcription factor, Twist1 upregulates genes essential for fibroblast activation^9,10^. In addition, the widespread expression of Twist1 in tumor stroma indirectly suggests its role in activated CAFs^10–13^. Moreover, the induction of Twist1 expression alone converts NFs into CAFs, and the downregulation of Twist1 impairs the pro-tumorigenic property of CAFs. These findings strongly suggest that Twist1 expression is necessary and sufficient for CAF activation^7^.

On the other hand, the suppression of CAF and tumor stroma has been investigated as a means of inhibiting tumor progression. Promising agents inhibiting CAF and tumor stroma formation have been reported in preclinical studies, but most of them have failed in clinical trials^14,15^. Moreover, suppressing CAFs by deleting α-SMA-positive myofibroblasts unexpectedly promoted tumor progression and reduced survival in a mouse pancreatic cancer model^16^, indicating the complicated involvement of CAFs in tumor progression. A new approach has been proposed to inhibit tumor growth by reversing tumor stroma into normal tissue^17^. Because NFs, unlike CAFs, create a tumor-restrictive environment, it is conceivable that deactivating CAFs could reverse a tumor-supportive microenvironment. Because we found Twist1 to be a novel marker that distinguishes CAFs from NFs, we developed a novel drug screening system using Twist1 as a marker to search for new candidate drugs that deactivate CAFs. In this drug screening procedure, Twist1 promoter-induced GFP was used as a cardinal indicator reflecting the activation of CAFs because Twist1 is a key inducer of CAFs. Using our system to screen for natural compounds that repress Twist1 promoter-induced GFP expression, we identified dihydrorotenone (DHR) as a CAF targeting compound. Our data demonstrate that DHR treatment inactivated CAFs and caused them to lose their tumor-promoting activity both *in vitro* and *in vivo*.

## Results

### HTS of a natural compound library using Twist1-promoter activity as a CAF-activation marker identifies dihydrorotenone as a novel candidate targeting CAFs

**Figure 1:**
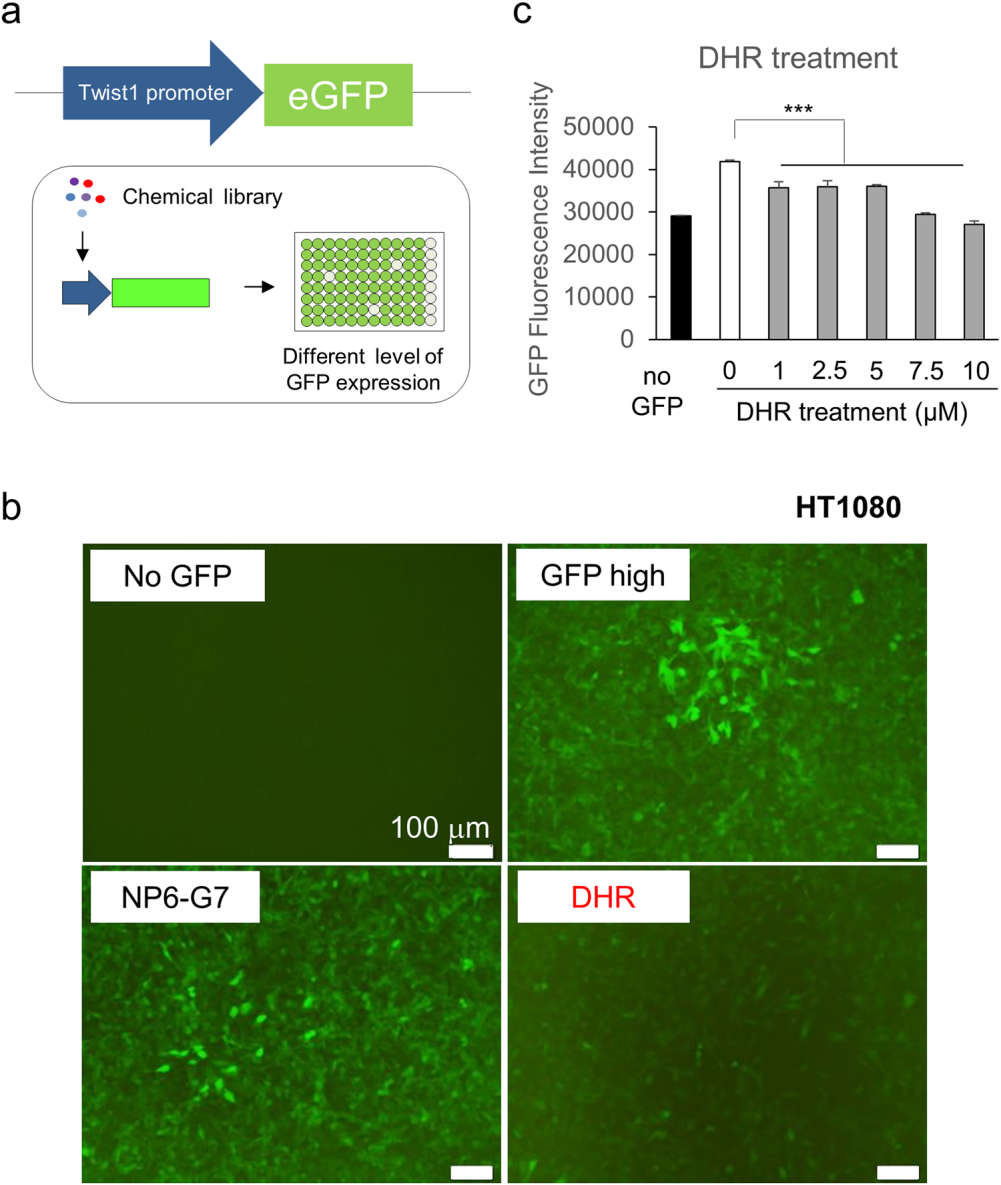
High-throughput screening of a natural compound library identifies dihydrorotenone as a novel candidate downregulating the expression of the CAF-enriched marker Twist1-GFP. (**a**) Illustration of the high-throughput screening of natural compounds downregulating Twist1 expression. 1.2kb Twist1 promoter-GFP was lentivirally delivered to HT1080 cells. As the Twist1 reporter cell line, HT1080 cells expressing GFP under the control of 1.2 kb Twist1 promoter were plated into 96-well cell culture plates and treated with the indicated compounds for three days. Efficacy was evaluated using the mean GFP fluorescence intensity. Compounds that showed less than 80% of GFP fluorescence intensity compared with control were selected as potent Twist1 downregulating drugs. (**b**,**c**) Validation of DHR’s efficacy in downregulating GFP expression under the control of 1.2 kb Twist1 promoter. (**b**) GFP expression of HT1080 cells treated with 1 μM NP6-G7 or DHR for three days. Selected compounds from the high-throughput screening, NP6-G7 and DHR, were separately administered to HT1080-Twist1 promoter GFP cells, and the GFP expression was monitored. HT1080-WT cells were used as the negative control. (**c**) GFP fluorescence intensity of HT1080-Twist1 promoter GFP cells treated with different concentrations of DHR. All experiments were done in triplicate. Bars represent the means ± SEM of three independent experiments.

For HTS, HT1080 cells were plated into 96-well plates and treated with a library of natural compounds for three days, and then the GFP fluorescence was measured (Fig. 1a). After several rounds of screening, six drug candidates that inhibited Twist1 promoter activity were selected from a preliminary screen of 87 compounds: DHR, andirobin, resveratrol 4′-methyl ether, antimycin A, camptothecin, and abscisic acid. Further experiments demonstrated that DHR significantly reduced GFP expression, identifying DHR as a potent drug candidate for deactivating CAFs (Fig. 1b,c).

**Figure 2:**
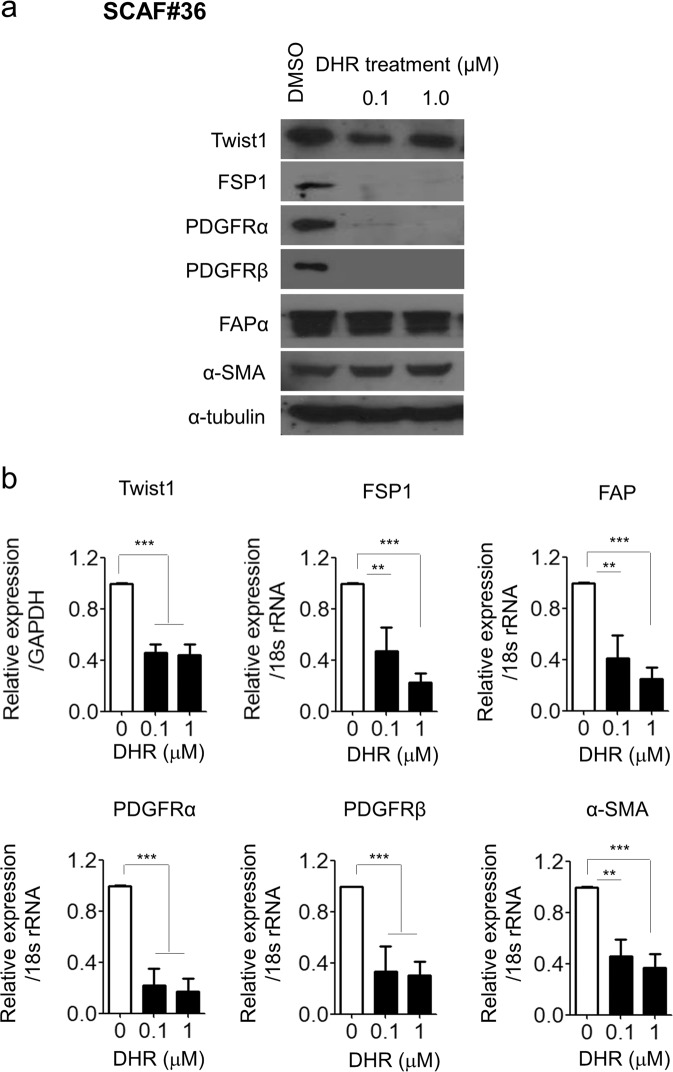
DHR treatment downregulates the expression of Twist1 and CAF markers. *In vitro* validation of DHR efficacy on cancer-associated fibroblasts, SCAF#36. (**a**) Western blotting analysis of Twist1, FSP1, PDGFRα, PDGFRβ, FAPα, and α-SMA after two weeks of treatment with 0.1 μM or 1 μM DHR. SCAF#36 cells were plated and treated with DHR for two weeks in medium containing 1% serum. α-Tubulin was used as a loading control. (**b**) Expression of transcripts for Twist1, FSP1, PDGFRα, PDGFRβ, FAPα, and α-SMA in SCAF#36 cells after three days of treatment with 0.1 μM or 1 μM DHR. Expression of Twist1 was normalized to GAPDH levels, and the other CAF markers were normalized to 18 s rRNA levels. Experiments were done in triplicate. Bars represent the means ± SEM of six independent experiments. Differences were evaluated by two-tailed student’s t-test. *P < 0.05; **P < 0.01; ***P < 0.005.

To confirm the CAF-deactivating effect of DHR, CAFs derived from stomach cancer tissue (SCAF#36) were treated with DHR for two weeks, and then the expression of several CAF markers was analyzed. As expected, DHR treatment decreased Twist1 protein levels compared with control SCAF#36 cells. Furthermore, DHR reduced the protein levels of well-known activated fibroblast markers such as FSP1, PDGFRα, and PDGFRβ (Fig. 2a). To determine the mRNA transcript expression level of the CAF-enriched markers, SCAF#36 cells were treated with DHR for three days, and then RNA was extracted for a qPCR analysis. As with the protein expression, DHR treatment decreased mRNA transcript levels of Twist1, FSP1, FAP, PDGFRα, PDGFRβ, and α-SMA compared with the control treatment (Fig. 2b). Thus, DHR decreased both the mRNA and protein expression of several key CAF-enriched markers.

### DHR induces CAF to switch from activated phenotypes to quiescent and inactive states

**Figure 3:**
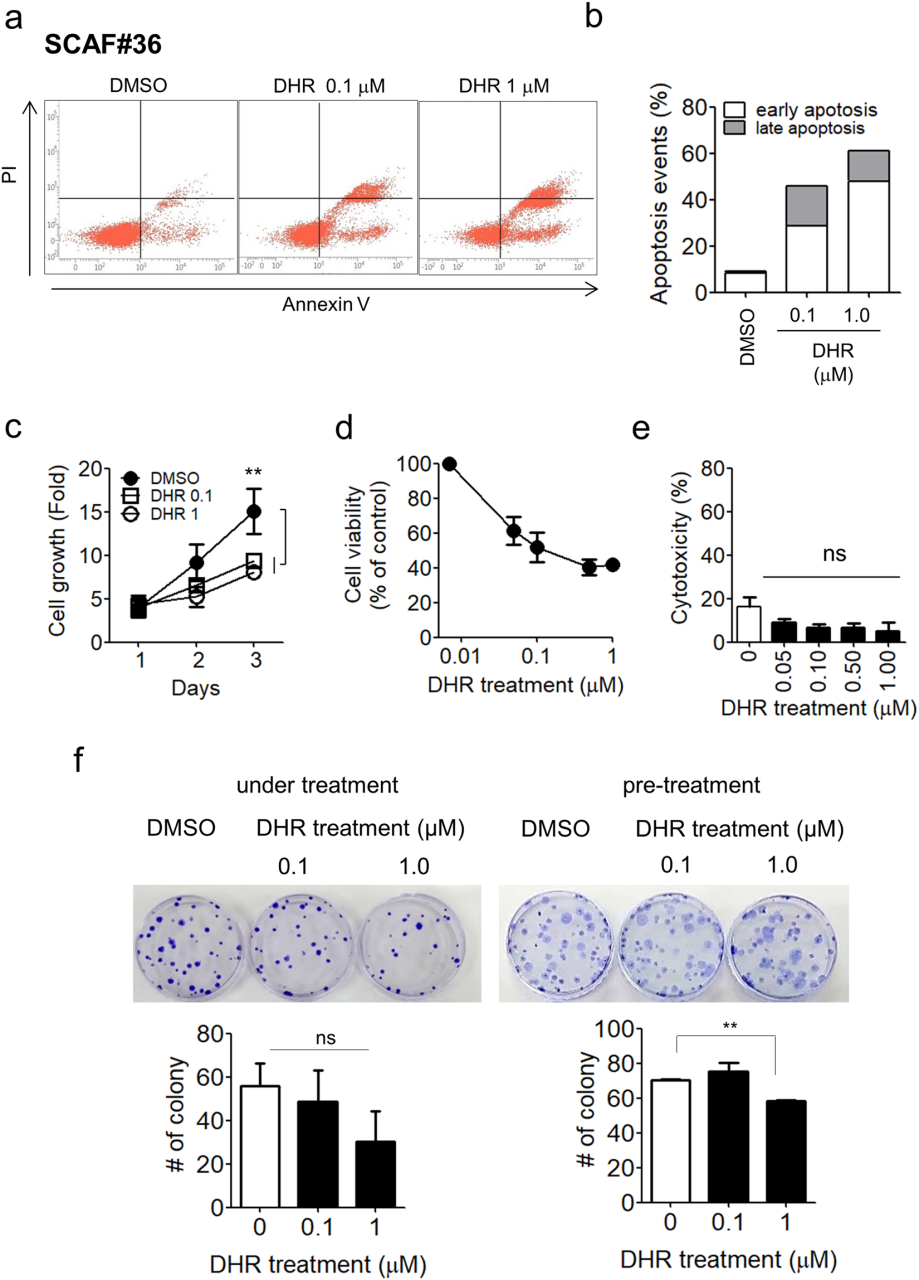
DHR treatment shifts CAFs into quiescent fibroblasts. (**a**,**b**) Flow cytometry analysis of apoptotic cell death in SCAF#36 cells after two weeks of 0.1 μM or 1 μM DHR treatment in medium containing 1% serum. (**a**) Treated cells were stained with annexin V-APC/propidium iodide, and apoptotic cell death was analyzed by flow cytometry. (**b**) Cells that underwent early apoptotic cell death (lower right quadrant) and late apoptotic cell death (upper right quadrant) were calculated as a percentage of all cells. (**c–e**) SCAF#36 cell proliferation, viability, and cytotoxicity under three days of 0.1 μM or 1 μM DHR treatment. (**c**) SCAF#36 cells were treated with 0.1 μM or 1 μM DHR, and cell proliferation was counted daily. Cell growth was normalized to day 0. Symbols represent the means ± SEM of five independent experiments. Differences were evaluated by two-tailed student’s t-test. **P < 0.01. (**d**) After 72 hours of DHR treatment, cell viability was assessed using an EZ-cytox kit. The optical absorbance measurement of formazan at 450 nm was assumed to be proportional to the number of viable cells in each well. Symbols represent the means ± SEM of three independent experiments. (**e**) The supernatants from SCAF#36 cells treated with DHR for 72 hours were assessed for LDH release with an EZ-LDH cytotoxicity assay kit. Experiments were done in triplicate. Bars represent the means ± SEM of three independent experiments. Differences were evaluated by two-tailed student’s t-test. (**f**) The effects of DHR treatment on the colonization of CAFs. SCAF#36 cells were plated, and the number of colonies was counted. Cells were either treated with 0.1 μM or 1 μM DHR every three days during colonization for two weeks (**left panels**) or pretreated for three days before colonization (**right panels**). Representative images are shown. Experiments were done in triplicate. Bars represent the means ± SEM of three independent experiments (treatment) or two independent experiments (pretreatment). Differences were evaluated by two-tailed student’s t-test. **P < 0.01.

Our finding that DHR decreased the expression of CAF markers led us to explore whether DHR functionally deactivated CAFs into a quiescent state. CAFs are known to be more proliferative and less prone to apoptosis than their normal counterparts^18^. To investigate whether DHR changed the induction of apoptosis, SCAF#36 cells were subjected to treatment with DHR for two weeks in medium containing 1% serum. Compared with the control, an increase in the percentage of apoptotic cells was observed in the DHR-treated cells (9% cell death in control cells vs. 45% cell death in 0.1 μM DHR-treated cells and 60% cell death in 1 μM DHR-treated cells), indicating that DHR augmented apoptosis (Fig. 3a,b). Next, we evaluated the effect of DHR treatment on cell proliferation. SCAF#36 cells were treated with DHR, and cell growth was evaluated each day by counting the number of cells. The total increase in the number of control cells was higher than in the DHR-treated cells after three days of treatment (more than a 15-fold increase for control cells versus an 9.3-fold increase for 0.1 μM DHR-treated cells and a 8-fold increase for 1 μM DHR-treated cells) (Fig. 3c). DHR treatment also reduced the proliferation of other stomach CAFs: SCAF#14, SCAF#32, and SCAF#39 (Supplementary Fig. 2a). To distinguish the cell growth inhibition effects of DHR from cytotoxic cell death, the LDH-based cytotoxicity assay was performed. After 72 hours of DHR treatment, the number of viable cells decreased in a concentration-dependent manner (Fig. 3d, Supplementary Fig. 2b). However, cytotoxic cell death did not change significantly (Fig. 3e, Supplementary Fig. 2c), indicating that the reduction in cell numbers with DHR treatment was due to cell-growth inhibition. To validate the specificity of DHR for inhibiting the growth of CAFs, stomach normal fibroblasts (SNF#32) were treated with various concentrations of DHR. Under normal conditions, SNF#32 showed less proliferation than several SCAF lines (more than a 10-fold increase for SCAFs versus less than a 5-fold increase for SNF#32, Supplementary Fig. 2a and 3b). Unlike the SCAF lines, growth inhibition was not observed in SNF#32 cells treated with 0.1 μM DHR, and it was only slightly reduced following treatment with 1 μM DHR (Supplementary Fig. 3a,b), suggesting that DHR treatment deactivated the highly proliferative CAFs into a more quiescent state.

Given those results, we hypothesized that DHR treatment would reduce clonogenic activity by deactivating CAFs. To test that hypothesis, we plated SCAF#36 cells and incubated them for two weeks. In CAFs treated with DHR every three days for two weeks, the number of colonies decreased strikingly compared with the control cells (Fig. 3f, left), indicating that DHR reduces the clonogenic activity of CAFs. We next examined whether DHR could have a long-term effect on the clonogenic ability of CAFs. SCAF#36 cells were treated with DHR for three days, and then they were plated for two weeks to determine colony formation. As seen in Fig. 3f, the number of colonies in cells treated with 1 μM DHR was significantly reduced (Fig. 3f, right). The number of colonies in cells treated with 0.1 μM DHR did not change; therefore, the deactivation of CAFs was somewhat reversible in low concentrations of DHR. Taken together, these findings suggest that CAFs switch from an activated phenotype into a quiescent phenotype following DHR treatment.

### DHR treatment represses ECM remodeling and cytokine production of CAFs

**Figure 4:**
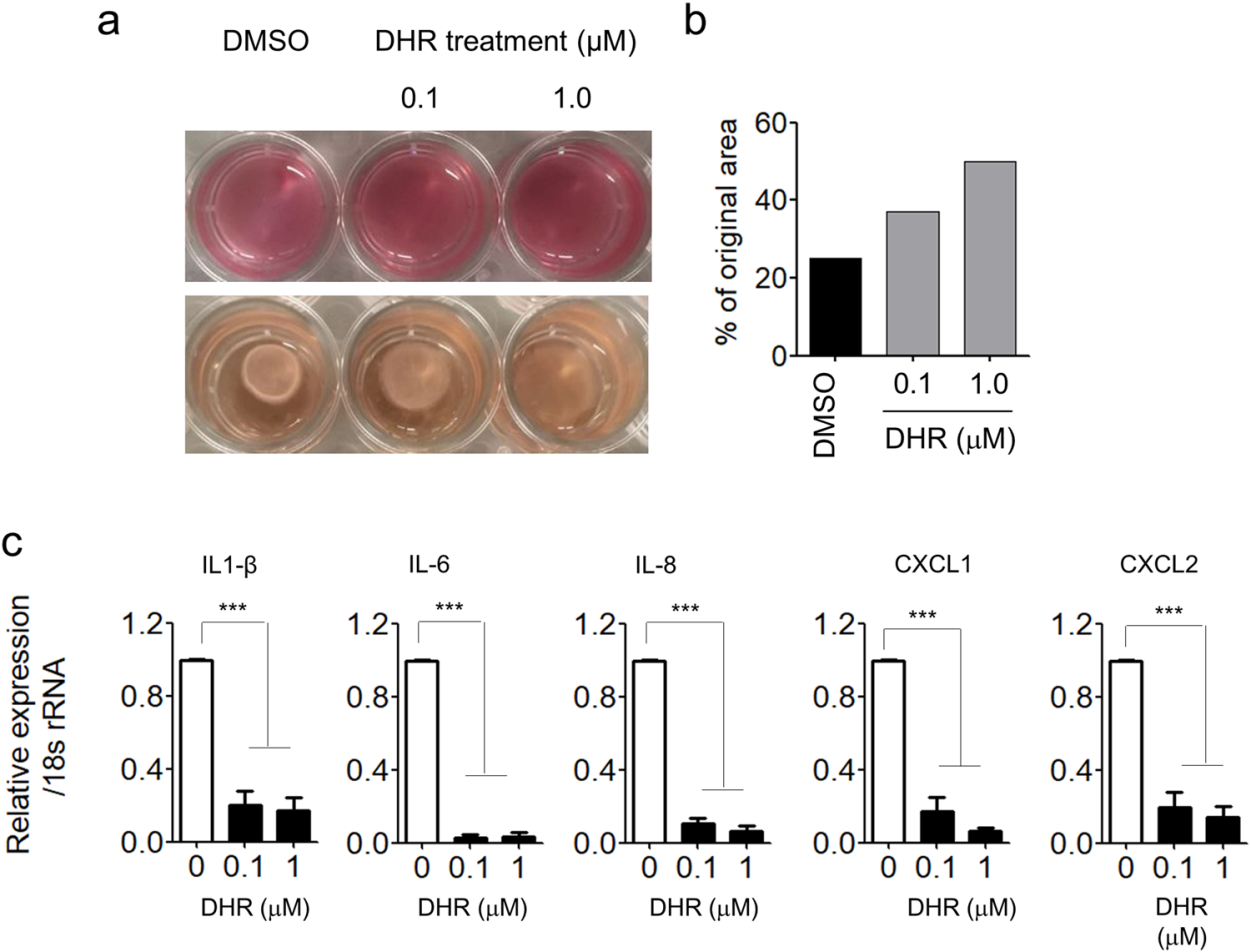
DHR treatment represses ECM remodeling and cytokine expression of CAFs. (**a–b**) Collagen gel remodeling capacity of SCAF#36 cells treated with 0.1 μM or 1 μM DHR for three days. (**a**) Representative images of collagen gel contraction. (**b**) Gel size is expressed as a percentage of the initial size. Similar results were observed in independent experiments. (**c**) Pro-inflammatory gene signatures of DHR-treated SCAF#36 cells. mRNA was isolated from SCAF#36 cells treated with 0.1 μM or 1 μM DHR for three days, and pro-inflammatory gene expression was normalized to 18 s rRNA levels. Experiments were done in triplicate. Bars represent the means ± SEM of six independent experiments. Differences were evaluated by two-tailed student’s t-test. ***P < 0.005.

Two major functions of CAFs are (1) ECM remodeling, which we assessed by collagen gel contraction, and (2) pro-inflammatory cytokine secretion^19,20^. The findings just described prompted us to test whether DHR can repress those major functions of CAFs. As shown in Fig. 4a,b, DHR-treated SCAF#36 cells showed markedly reduced abilities in contracting collagen gel matrices (75% contraction for control cells vs. 63% contraction for 0.1 μM DHR-treated cells, and 50% contraction for 1 μM DHR-treated cells), indicating a reduction in the ECM remodeling ability of CAFs. To further assess the effect of DHR on pro-inflammatory cytokine gene expression in CAFs, several pro-inflammatory gene signatures were accessed by real-time PCR. Consistent with previously published findings^19,20^, DHR-induced a significant downregulation of IL1-β, IL-6, IL-8, CXCL1, and CXCL2 (Fig. 4c).

### DHR treatment suppresses the tumor-promoting abilities of CAFs

**Figure 5:**
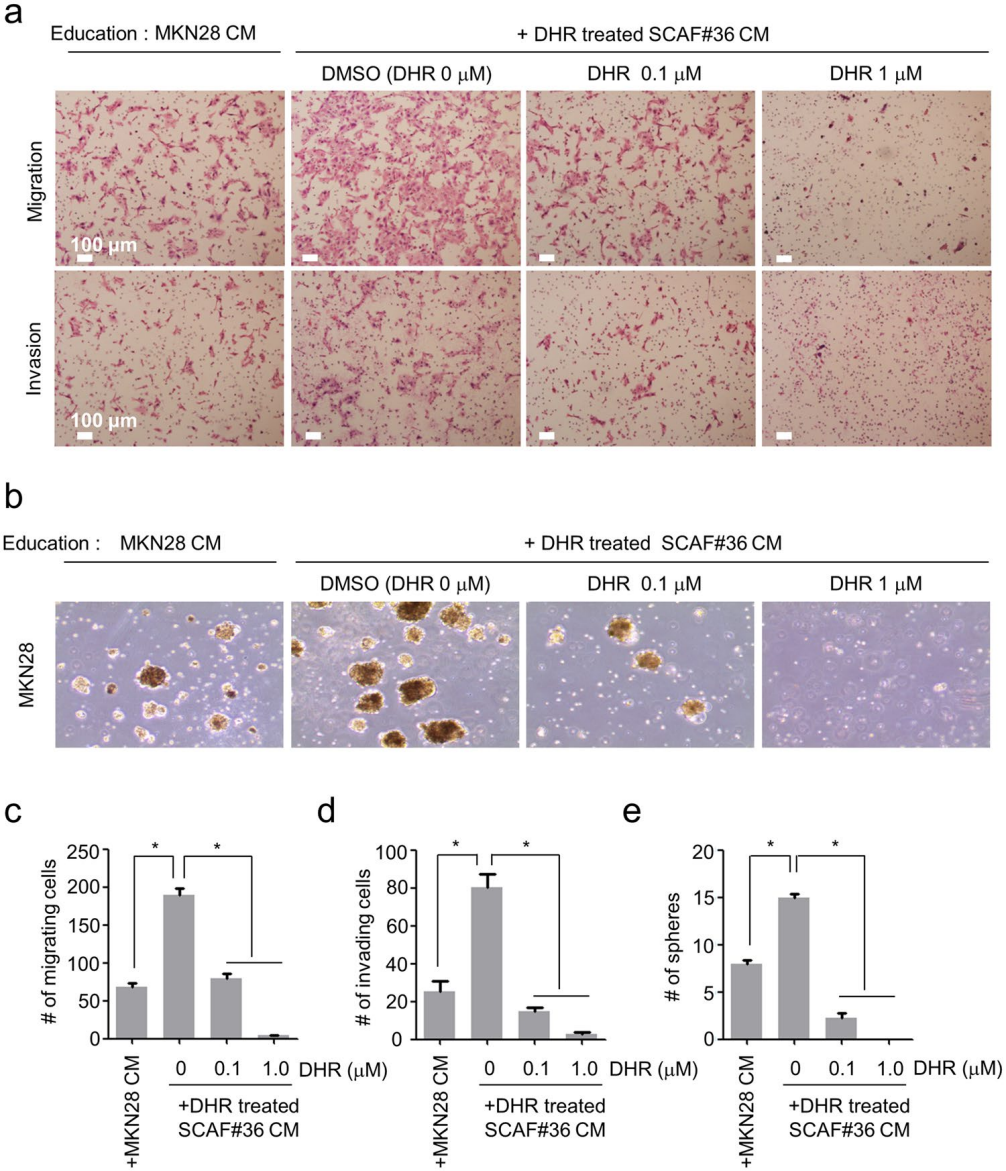
DHR treatment suppresses the tumor-promoting ability of CAFs. MKN28 human stomach cancer cells were incubated with appropriate conditioned medium for two days and analyzed to assess their capacity for migration, invasion, and tumorsphere formation. Conditioned media were prepared from SCAF#36 cells treated with DMSO or DHR for three days. Conditioned medium collected from MKN28 cells was used as the control. (**a**, **c,d**) Migration and invasion of MKN28 cells treated with conditioned medium from SCAF#36 cells. Representative photomicrography of migration (**upper panels**) and invasion (**lower panels**) of treated MKN28 cells (**a**). The numbers of migrating (**c**) and invading (**d**) cancer cells were quantified. (**b**, **e**) Tumorsphere formation of MKN28 cells treated with conditioned medium from SCAF#36 cells. Representative photomicrography (**b**) and quantification (**e**) of MKN28 tumorspheres. Scale bar, 100 μm. Experiments were done in triplicate. Bars represent the means ± SEM of two independent experiments. Differences were evaluated by two-tailed student’s t-test. *P < 0.05.

CAFs have been reported to promote tumor growth and invasion by secreting paracrine factors^21–23^, remodeling ECM^22^, and developing vascular networks^5,24^. Several studies have reported that soluble cytokines and pro-inflammatory signals from CAFs can directly stimulate tumor cell proliferation^25^. Based on our findings that DHR treatment reduces pro-inflammatory gene signatures, we hypothesized that DHR would suppress the tumor-supporting ability of CAFs. To test that hypothesis, we established an indirect co-culture model by incubating human stomach cancer cell lines with conditioned media prepared from SCAFs. As expected, incubation with conditioned medium from SCAF#36 cells increased MKN28 cancer cell migration and invasion compared with cells incubated with conditioned medium from other MKN28 cells (more than 3-fold increase in migrating cells and slightly less than 4-fold increase in invading cells), indicating the tumor-supporting effects of CAFs (Fig. 5a,c,d). We then tested our hypothesis using conditioned medium prepared from DHR-treated SCAF#36 cells. In agreement with our hypothesis, the SCAF#36 cell-conditioned medium-induced enhancement of migration and invasion in cancer cells was significantly diminished by DHR treatment, suggesting that the pro-tumorigenic capacity of CAFs was eliminated by DHR (Fig. 5a,c,d). These experiments were repeated using additional stomach cancer cells and stomach CAFs. Specifically, conditioned medium from DHR-treated SCAF#14, SCAF#36, or SCAF#39 cells inhibited the migration and invasion of both MKN28 cancer cells (Supplementary Fig. 4a,b) and SNU668 cancer cells (Supplementary Fig. 5a–c). Furthermore, we evaluated the direct influence of DHR on MKN28 and SNU668 cancer cells to rule out the possibility of DHR carryover contamination and investigate the direct effect of DHR on cancer cells. DHR treatment reduced the proliferation of both MKN28 cells and SNU668 cells in a dose dependent manner (Supplementary Fig. 6a and 7a). DHR-treated MKN28 cancer cells showed inhibited migration and invasion compared with control cells (Supplementary Fig. 6b). However, decreased migration and invasion of SNU668 cancer cells were shown only with 1 μM DHR treatment (Supplementary Fig. 7b). In addition, to evaluate the effects of potential carryover contamination of DHR in conditioned medium, MKN28 cancer cells and SNU668 cancer cells were subjected to conditioned medium prepared from DHR-treated cancer cells. Interestingly, the migration ability of MKN28 (Supplementary Fig. 6c) and SNU668 cancer cells (Supplementary Fig. 7c) was not abolished under conditioned medium collected from DHR-treated MKN28 cells or SNU668 cells, suggesting that the suppressed migration and invasion of cancer cells under conditioned medium from DHR-treated SCAFs were not caused by carryover contamination of DHR.

In addition to enhancing the migration and invasion of cancer cells, CAFs have been known to increase tumorsphere formation, indicating the enrichment of the cancer stem-cell population^2^. As a next step, we studied the effect of DHR treatment on CAF-induced enhancement of tumorsphere formation. Prior to the analysis, MKN28 cells were incubated with conditioned medium collected from MKN28 or SCAF#36 cells and then grown in serum-free non-adherent conditions for two weeks. Consistent with the known cancer stem cell–enhancing effects of CAFs, the number of tumorspheres increased when MKN28 cells were incubated with conditioned medium from SCAF#36 cells. We then treated SCAF#36 cells with DHR and repeated the sphere formation assay. In line with our hypothesis, the SCAF#36-induced enhancement of tumorsphere formation was greatly reduced by treating the SCAF#36 cells with DHR (Fig. 5b,e). Taken together, the pro-tumorigenic functions of CAFs were markedly decreased by DHR treatment.

### DHR impairs CAF-induced tumor growth-promotion *in vivo*

**Figure 6:**
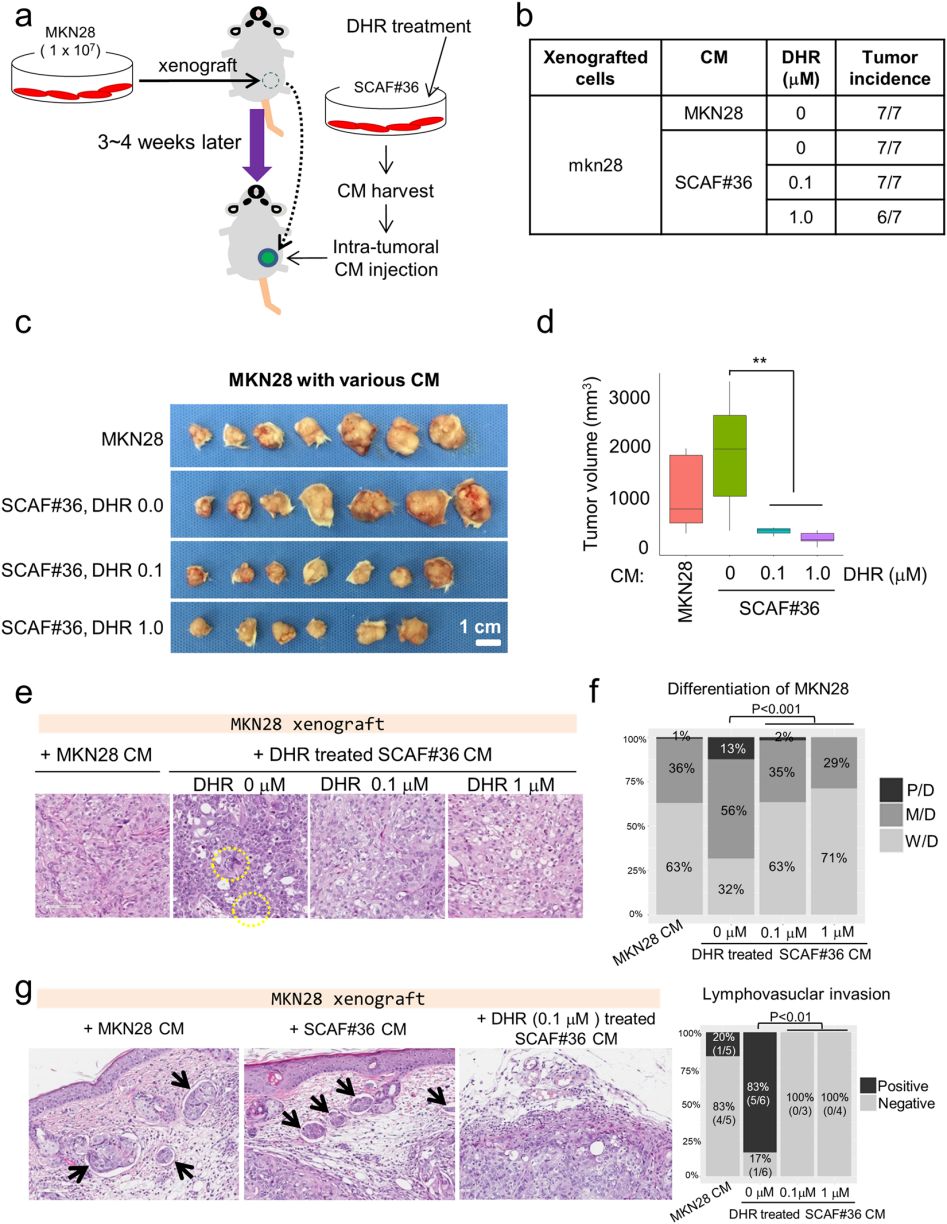
DHR treatment attenuates tumor promoting ability of CAFs in xenografts of MKN28 cells. (**a**) Illustration of experimental scheme. MKN28 cells (1*10^6^) were subcutaneously injected into the flanks of NOD/SCID mice. When the tumors were palpable, mice (n = 7 per group) were treated with appropriate conditioned medium every three days by intratumoral injection. Conditioned media were collected from MKN28, SCAF#36, and SCAF#36 treated with 0.1 μM or 1 μM DHR for three days. (**b**) The numbers of developed tumors in the indicated treatment conditions. Photographs of gross tumors (**c**), tumor volume (**d**), hematoxylin and eosin staining (20× magnification) of representative tumors (**e**), quantification of tumor grade as well differentiated (WD), moderately differentiated (MD), and poorly differentiated (PD) (**f**), and quantification of lymphovascular invasion (**g**). Whiskers represent the mean with min to max in (**d**). Differences were evaluated using Tukey’s honestly significant difference test. **P < 0.01. Note that there was no endolymphatic tumor emboli in tumors injected with conditioned medium from DHR-treated SCAF#36 cells.

Given the *in vitro* evidence that DHR reduces the tumor-supporting functions of CAFs, we next studied the *in vivo* effect of DHR on the tumor-supportive efficacy of CAFs by investigating the effects of conditioned medium from SCAF#36 cells in a mouse tumor-xenograft model. For this test, MKN28 cells were inoculated into the flanks of NOD/SCID mice. When the tumors were palpable, the mice were randomly assigned to one of 4 groups (MKN28-CM, SCAF#36-CM, SCAF#36-DHR 0.1 μM-CM, and SCAF#36-DHR 1 μM-CM), and treated with the appropriate conditioned medium every three days by intratumoral injection (Fig. 6a). Tumorigenesis was remarkably enhanced when the tumors were treated with conditioned medium from SCAF#36 cells compared with MKN28 cells, validating the tumor-promoting ability of CAFs (average tumor volume: 1095.5 ± 766.4 in MKN28-CM vs. 1489.3 ± 1097.2 in SCAF#36-CM) (Fig. 6b–d). Mice treated with conditioned medium derived from DHR-treated SCAF#36 cells showed significant suppression in tumor growth in a dose-dependent manner compared with control mice treated with conditioned medium derived from DMSO-treated SCAF#36 cells (Fig. 6b–d). Interestingly, the tumors in the group treated with conditioned medium from MKN28 cells were well to moderately differentiated (Fig. 6e,f). However, the tumors treated with conditioned medium from SCAF#36 cells showed more poorly differentiated giant cells, endolymphatic tumor emboli, and perineural invasion, which indicates rapid tumor growth (Fig. 6g). The tumors treated with conditioned medium from SCAF#36 cells showed enhanced expression of Twist1 and α-SMA (Supplementary Fig. 8a). In addition, immunostaining of Ki-67 and cleaved caspase-3 demonstrated an increase of cell proliferation and decrease of cell death in tumors treated with conditioned medium from SCAF#36 (Supplementary Fig. 8a–c). These pro-tumorigenic effects were diminished when conditioned medium collected from SCAF#36 cells treated with DHR was administered (Fig. 6e–g, Supplementary Fig. 8a–c). Notably, no metastasis into the lung or liver was found (data not shown). Consistent with the *in vitro* findings, our *in vivo* data indicate that soluble factors from SCAF#36 cells trigger the proliferation and dedifferentiation of cancer cells, and those effects were attenuated by DHR treatment, which led to the suppression of tumor growth.

### Deactivation of CAFs by DHR was partially dependent on Twist1 expression

**Figure 7:**
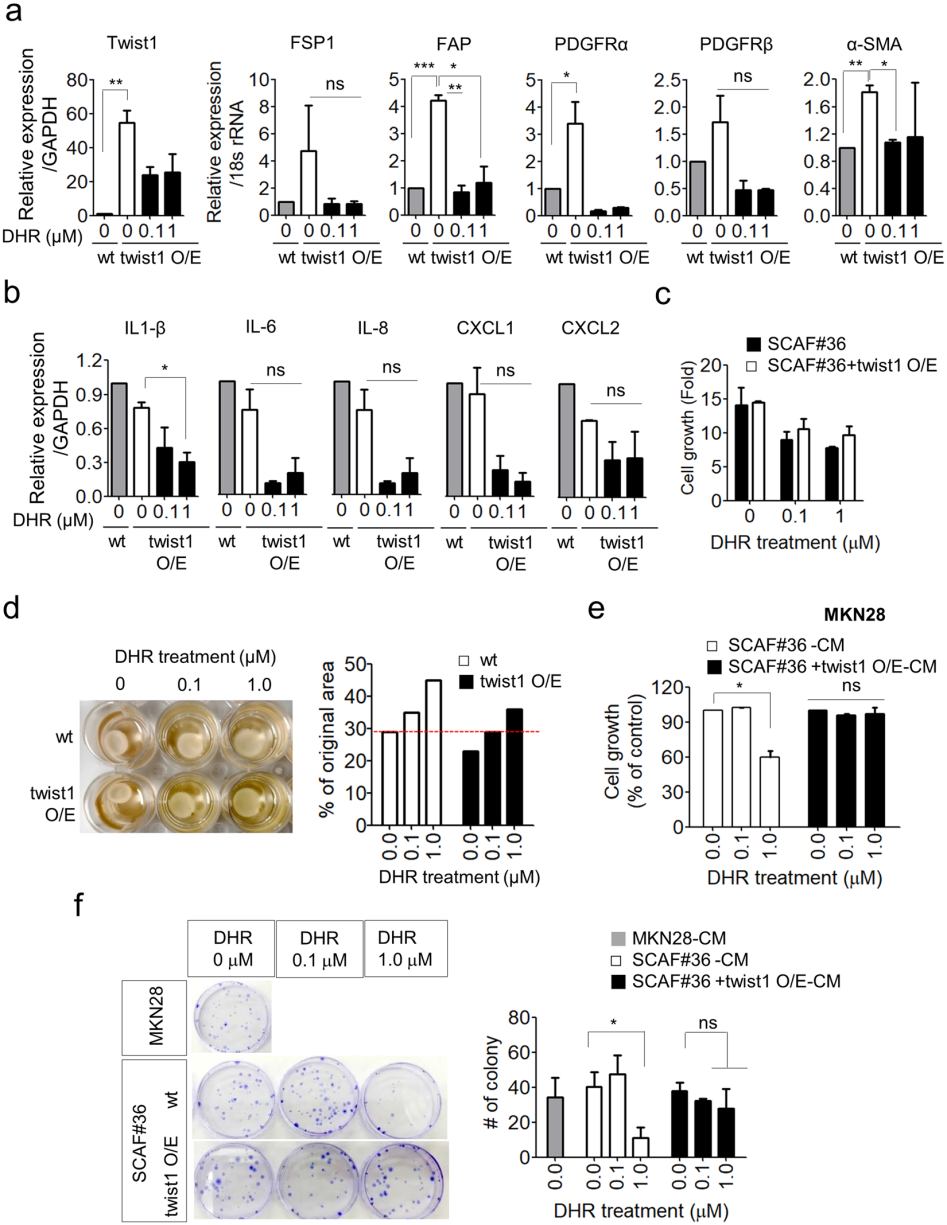
Forced expression of Twist1 partially restores the functional activity of CAFs under DHR treatment. (**a-d**) Gene signature, proliferation and gel remodeling capacity of SCAF#36-Twist1 O/E cells treated with 0.1 μM or 1 μM DHR for three days. Differences were evaluated by two-tailed student’s t-test. *P < 0.05; **P < 0.01; ***P < 0.005. (**a**) Expression of transcripts for Twist1, FSP1, PDGFRα, PDGFRβ, FAPα, and α-SMA in SCAF#36-Twist1 O/E cells. SCAF#36-wt cells were used as a control. Expression of Twist1 was normalized to GAPDH levels and the other CAF markers were normalized to 18 s rRNA levels. Experiments were done in triplicate. Bars represent the means ± SEM of two independent experiments. (**b**) Pro-inflammatory gene signatures of DHR treated SCAF#36-Twist1 O/E cells. Pro-inflammatory gene expression was normalized to 18 s rRNA levels. SCAF#36-wt cells were used as a control. Experiments were done in triplicate. Bars represent the means ± SEM of two independent experiments. (**c**) Proliferation of SCAF#36 or SCAF#36-Twist1 O/E cell under DHR treatment. Cell growth was normalized to day 0. Bars represent the means ± SEM of three independent experiments. (**d**) Collagen gel remodeling capacity of DHR-treated SCAF#36-Twist1 O/E cells. Gel size is expressed as a percentage of the initial size. Similar results were observed in independent experiments. (**e-f**) Proliferation and colony formation of MKN28 cells incubated with conditioned medium collected from SCAF#36-wt or SCAF#36-Twist1 O/E cells. Differences were evaluated by two- tailed student’s t-test. *P < 0.05. (**e**) MKN28 cells were incubated with appropriate conditioned medium for two days and then cultured for three days. Cell growth was assessed using an EZ-cytox kit and normalized to the cells incubated with conditioned medium without DHR treatment. Experiments were done in triplicate. Bars represent the means ± SEM of two independent experiments. (**f**) MKN28 cells were incubated with appropriate conditioned medium for two days and then cultured for colony formation. Representative images are shown. Experiments were done in triplicate. Bars represent the means ± SEM of three independent experiments.

Because DHR was initially selected for testing because it decreased GFP expression under the control of a Twist1-promoter, we explored whether the effect of DHR on CAFs was mediated by Twist1 downregulation. To address this issue, SCAF#36 cells constitutively overexpressing Twist1 by means of a CMV promoter were treated with DHR and analyzed for CAF marker expression. Twist1 overexpression increased the levels of CAF markers in accordance with a previous report^7^, indicating that Twist1 is a CAF regulator (Fig. 7a). However, Twist1 overexpression failed to restore the expression of CAF markers decreased by DHR treatment (Fig. 7a). Moreover, DHR-induced changes in the pro-inflammatory gene expression of CAFs were not restored by Twist1 overexpression either (Fig. 7b).

In contrast to the expression of CAF markers and pro-inflammatory cytokine genes, the DHR-induced suppression of CAFs’ proliferation and gel contracting capacities was partially restored by constitutive Twist1 overexpression. Specifically, constitutive overexpression of Twist1 in SCAF#36 cells partially restored DHR-induced suppression of CAF proliferation (Fig. 7c), although the difference was marginal. Moreover, SCAF#36 cells overexpressing Twist1 exhibited better collagen contraction, even under DHR treatment (Fig. 7d), further suggesting the possibility that Twist1 expression could rescue the effects of DHR on SCAF#36 cells, at least partially.

Next, the effect of constitutive Twist1 overexpression on the DHR-induced suppression of CAF tumor-supporting capacity was evaluated by indirect co-culture experiments using conditioned medium from SCAF#36-WT or SCAF#36-Twist1 O/E(overexpression) cells. MKN28 cells were treated with the respective conditioned media for 48 hours, then plated and cultured for three days. In contrast to the reduced proliferation of MKN28 cells incubated with conditioned medium from 1 μM DHR-treated SCAF#36-WT, the growth of MKN28 cells was not inhibited by conditioned medium from DHR-treated SCAF#36-Twist1 O/E, indicating that constitutive Twist1 overexpression successfully restored the tumor-supporting capacity of CAFs (Fig. 7e). Furthermore, MKN28 cells incubated with conditioned medium collected from SCAF#36-Twist1 O/E maintained colony formation without any significant decrease despite treating the CAFs with DHR (Fig. 7e). In contrast, MKN28 cells showed a significant reduction in colony formation upon incubation with conditioned medium from SCAF#36-WT treated with 1 μM DHR (Fig. 7f), suggesting that the DHR suppression of tumor-promoting capacity was neutralized by Twist1 overexpression in CAFs.

Taken together, our results indicate that constitutive Twist1 overexpression partially restores the tumor-promoting capacity of CAFs under DHR treatment, suggesting that the effects of DHR are partially dependent on Twist1 expression.

## Discussion

CAFs, the major component of tumor stroma, are known to generate a tumor-prone microenvironment by secreting paracrine factors and ECM components to activate cancer cell survival and metastasis^1^. Based on CAFs’ positive influence on tumor cell behavior, recent anticancer therapies have focused on modulating tumor stroma^14,26^.

Despite the urgent needs for an anti-CAF drug, no effective and feasible drug screening system has been specialized for anti-CAF drug discovery. The biggest obstacle to the development of such a drug screening system is the lack of simple, fast, and accurate assays to evaluate the effects of drugs on CAFs. In this study, we attempted to overcome this problem by developing an assay method that can easily measure CAF activity using fluorescence. Specifically, we developed a reporter system that expresses GFP under the control of a Twist1 promoter. Because Twist1 is a key transcription factor that transforms normal, inactive fibroblasts into CAFs, this reporter system reflects CAF activity well. To use this reporter system for HTS, we built a drug screening system for anti-CAF drugs and demonstrated through this study that it works well.

DHR is a natural pesticide that binds to electron transport chain complex I, inhibits mitochondrial function, and disrupts ATP production^27^. Even though DHR is not considered to be toxic, it induces toxicities in several experimental models, such as apoptosis of human plasma cells^27^, cell-cycle arrest in plasma cancer cells^28^, and Parkinson’s syndrome in rats treated with DHR long-term^29,30^. However, the role of DHR in regulating the activity of cells has not been elucidated. Here, we found that DHR, which decreased Twist1 promoter-GFP expression, deactivated SCAF#36 cells, induced apoptosis, and inhibited growth. Because Twist1 promoter-GFP expression was used as a marker for CAF activation in this experimental setting, we tested whether decreased Twist1 expression was caused by CAF deactivation or just a reflection that CAFs had been inactivated by mitochondrial inhibition. Given the evidence that Twist1 regulates the expression of genes essential for fibroblast activation^7^, we hypothesized that impaired expression of Twist1 might deactivate CAFs. Our results show that the overexpression of Twist1 neutralized DHR’s inhibition of the functional activity of CAFs but not perfectly. These data suggest that the deactivation of CAFs by DHR was mediated by Twist1 downregulation but also acted through additional Twist1-independent mechanisms (Fig. 7).

It is possible that impaired mitochondrial function caused by DHR deactivated CAFs into more quiescent fibroblasts through metabolic reprograming, as suggested by studies showing that proper mitochondrial function is essential for deciding the fate of stem cells, such as proliferation and differentiation^31,32^. However, increasing evidence suggests that CAFs switch their metabolism from oxidative phosphorylation to aerobic glycolysis in response to signals from cancer cells^33^. Considering that activated CAFs rely mostly on glycolysis to meet their energy demands, as well as to provide the metabolic intermediates for cancer cells^34^, the inhibitory effect of DHR on oxidative phosphorylation might not account for the deactivation of CAFs. Instead, the oxidative stress in mitochondria might enhance the inactivation of CAFs.

In this study, we first identified DHR as an anti-CAF drug candidate using reporter cells expressing Twist1 promoter-GFP and confirmed that DHR repressed tumor progression both *in vitro* and *in vivo* by deactivating CAFs. Moreover, in accordance with our previous report showing the inactivation of CAFs by shRNA-mediated downregulation of Twist1^7^, we found that Twist1 downregulation was involved in DHR-induced CAF deactivation. To determine the degree of CAF activation, we used a Twist1 promoter-GFP reporter system. Considering its simplicity, our system is suitable for HTS and will accelerate the discovery of new anti-CAF drugs. Furthermore, the finding that Twist1 plays an essential role in the pathogenesis of pathological fibrosis indicates that DHR, a compound inhibiting Twist1 expression in fibroblasts, can be considered a potential therapeutic agent for fibrotic diseases in addition to modifying the cancer microenvironment.

## Materials and Methods

### Cell culture

Previously isolated human stomach CAFs #14, 32, 36, 39 (SCAF#14, SCAF#32, SCAF#36, and SCAF#39)^7^, stomach fibroblasts #32 (SNF#32), MKN28 stomach cancer cells (Korean Cell Line Bank, Seoul, Korea), and SNU668 stomach cancer cells (Korean Cell Line Bank, Seoul, Korea) were maintained in DMEM/F12 (1:1) medium supplemented with 10% FBS (Welgene, Gyeongsan, Korea) and 1% antibiotics (Life Technologies, Carlsbad, CA). HT1080 fibrosarcoma cells (Korean Cell Line Bank, Seoul, Korea) were cultured in RPMI1640 medium supplemented with 10% FBS (Welgene) and 1% antibiotics (Life Technologies, Carlsbad, CA).

SCAF#36 and HT1080 cells were lentivirally transduced using pLV-eGFP plasmids (a gift from Pantelis Tsoulfas, Addgene plasmid#36083) to express GFP under the control of 1.2 kb of human Twist1 promoter (−1043– +239 from transcription start site). The GFP high (Twist1+) cell population was sorted using FACS Aria III (BD Biosciences, Franklin Lakes, NJ) and used for all experiments. For the rescue experiment, SCAF#36 expressing GFP under the control of 1.2 kb Twist1 promoter was lentivirally transduced using pHR’CMV-Twist1 plasmids.

### High-throughput screening (HTS) for compounds deactivating CAF

For HTS to find drug candidates that can deactivate CAFs, human fibrosarcoma HT1080 cells expressing GFP under the control of 1.2 kb Twist1 promoter were used. HT1080 cells expressing GFP were plated into 96-well cell culture plates at a density of 5,000 cells per well and treated with 1 μM samples from a drug library of natural compounds for three days. Drug efficacy was measured as GFP fluorescence intensity using a multimode microplate reader (TECAN, Männedorf, Switzerland). To validate the six best drug candidates from the HTS, HT1080 cells were plated in 12-well plates at 20,000 cells per well and treated individually with those 6 compounds. Results from the assays were confirmed in each of three runs.

### Reagents and antibodies

Dihydrorotenone was provided by Dr. Kyeong Kyu Kim at Sungkyunkwan University (Suwon, Korea). An annexin-V staining kit was purchased from BD Bioscience (Franklin Lakes, NJ). An EZ-cytox cell viability assay kit was purchased from Daeill Lab (Seoul, Korea). A cell contraction assay kit was purchased from Cell Biolabs, Inc (San Diego, CA). Twist1 (Cat: ab50887, Abcam, Cambridge, MA), FSP1 (Cat: 07-2274, Millipore, Burlington, MA), PDGFRα (Cat: S3164, Cell Signaling, Danvers, MA), PDGFRβ (Cat: ab32570, Abcam, Cambridge, MA), FAPα (Cat: 53066, Abcam, Cambridge, MA), α-SMA (Cat: (E184) 04-1094, Millipore, Burlington, MA) and α-tubulin antibodies (Cat: SC-8035, Santa Cruz, Dallas, TX) were used for immunoblotting.

### Western blot analyses

SCAF#36 cells were treated with DHR for two weeks in DMEM/F12 (1:1) medium containing 1% FBS and 1% antibiotics. Cells were harvested and incubated on ice for 30 min in RIPA lysis buffer containing 50 mM Tris (pH 7.4), 150 mM NaCl, 1 mM EDTA, 1% Triton X-100, 1% Na-Doc, 0.1% SDS, and protease inhibitors (Roche, Basel, Switzerland). After centrifugation at 13,000 ×*g* for 10 min at 4 °C, the supernatants were used for immunoblotting to detect Twist1, FSP1, PDGFRα, PDGFRβ, FAPα, α-SMA, and α-tubulin.

### RNA purification and real-time RT-PCR

Total RNA was extracted from SCAF#36 cells treated with vehicle or DHR for 72 hours using Trizol (Cat: 15596026, ThermoFisher, Waltham, MA) according to the manufacturer’s instructions and quantified by OD_260_/OD_280_ measurement. cDNA was synthesized from 1 μg of total RNA using a high capacity cDNA reverse transcription kit (Cat: 4368813, Applied Biosystems, Foster City, CA). To detect different fibroblast markers, real-time RT-PCR was performed using SYBR green PCR mixture (Cat: 4309155, Applied Biosystems, Foster City, CA) with an ABI 7900 HT fast real-time PCR system. All experiments were performed in triplicate, and relative expression was normalized to GAPDH or 18 s rRNA. The primer sequences used are listed in the Supplementary Methods.

### Analysis of apoptosis

Apoptosis assays were performed using an annexin V-APC apoptosis detection it (Cat: 556547, BD Biosciences, Franklin Lakes, NJ) according to the manufacturer’s instructions. After two weeks of DHR treatment in DMEM/F12 (1:1) medium containing 1% FBS and 1% antibiotics, cells were harvested, washed twice with cold PBS, and then resuspended in annexin-V binding buffer. The cells were then stained with annexin-V conjugated with APC at room temperature in the dark for 30 min and subjected to flow cytometry as soon as possible (within 1 hour).

### Cell proliferation, viability, and cytotoxicity assays

To validate drug efficacy *in vitro*, SCAF#36 cells were seeded into 6-well plates at a density of 40,000 cells per well. The next day, cells were treated with DHR dissolved in DMEM/F12 medium supplemented with 10% FBS and 1% antibiotics. The effect of DHR on cell proliferation was assessed by counting the number of cells after trypan blue staining.

Cell viability and cytotoxicity were assessed using an EZ-cytox cell viability assay kit (Cat: EZ-100, DaeilLab, Seoul, Korea) and EZ-LDH cell cytotoxicity assay kit (Cat: DG-LDH1000, DoGen, Seoul, Korea). Assays were performed according to the manufacturers’ instructions. Briefly, SCAF#36 cells were plated into 96-well plates at a density of 5,000 cells per well, treated with DHR for 72 hours, and then assessed for cell viability and cytotoxicity.

### Colony formation assay

SCAF#36 cells were pretreated with DHR for three days or treated with DHR during a colony formation assay. SCAF#36 cells were plated into 60-mm plates in triplicate at a density of 100 cells per plate. Every three days, the medium was replaced, and fresh DHR was added. In the pretreated cells, the medium was replaced without DHR. After two weeks of culture, the cells were fixed in methanol for 5 minutes and stained with 0.1% crystal violet, and then the number of colonies was counted.

### Collagen gel contraction assay

A fibroblast contraction assay was performed using a cell contraction assay kit (Cat: CBA-201, Cell Biolabs, Inc, San Diego, CA) according to the manufacturer’s instructions. DHR- or vehicle-treated SCAF#36 cells were trypsinized and mixed with collagen. The cell-collagen mixture was plated into 24-well plates and incubated at 37 °C for 1 hour to allow collagen polymerization. Collagen gel contraction was initiated by releasing the gel from the side of the well, and changes in the collagen gel size were measured.

### Preparation of conditioned media

To collect conditioned media, cancer cells or SCAF cells were treated with or without DHR for 72 hours, washed three times with PBS, and then incubated with serum-free medium. After 24 hours of incubation, the conditioned media were harvested, centrifuged, and collected.

### Transwell migration and invasion assay

Invasion and migration assays were performed as previously described^7^. MKN28 human stomach cancer cells were incubated with appropriate fibroblast conditioned medium for two days, washed with PBS, and then re-suspended in a serum free medium containing 0.1% BSA (bovine serum albumin). Cells were plated into the upper chamber of a 24-well Transwell insert (Cat: 354234, Corning, Corning City, NY) at a density of 100,000 cells per well. Culture medium containing 10% serum was used as chemo-attractant. After 24 hours, cells were fixed with methanol for 5 minutes, and stained with hematoxylin. Cells from the upper compartment were removed, and the migrated/invaded cells were imaged with a microscope.

### *In vitro* tumorsphere formation assay

MKN28 cells were incubated with appropriate fibroblast conditioned medium for two days and then seeded into ultra-low-attachment 6-well plates (Cat: CLS3471, Corning, Corning City, NY) at a density of 50,000 cells per well. Cells were grown in serum-free DMEM/F12 (1:1) medium containing EGF (20 ng/ml), bFGF (20 ng/ml), and B27 supplements. After two weeks of incubation, tumorspheres were counted.

### *In vivo* drug efficacy evaluation using mouse xenograft model

To examine how DHR affected the tumor promotion activity of CAFs, 1 × 10^6^ MKN28 cells were subcutaneously inoculated into the flanks of nonobese diabetic-severe combined immunodeficiency (NOD/SCID) mice (OrientBio, Sungnam, Korea). At 3–4 weeks post xenograft, mice were randomly assigned to one of 4 groups (MKN28-CM, SCAF#36-CM, SCAF#36 treated with DHR 0.1 μM-CM, and SCAF#36 treated with DHR 1 μM-CM), and the appropriate conditioned medium was injected twice per week via intratumoral injection. The mice were sacrificed when the average tumor volume reached about 2,000 mm^3^, and the tumors were dissected. Tumor tissues were fixed in 10% neutral buffered formalin for 48 h and stained with H&E for pathological diagnoses. All animal experiments were approved by the Institutional Animal Care and Use Committee of Samsung Medical Center at Sungkyunkwan University and performed in accordance with the relevant guidelines and regulations.

## Supplementary information

Supplementary information.

## Data Availability

The datasets generated or analyzed during this study are included in this article (and its Supplementary information file).
