# Supplementary material for: New screening system using Twist1 promoter activity identifies dihydrorotenone as a potent drug targeting cancer-associated fibroblasts
Source: Sci Rep. 2020 Apr 27;10:7058. doi: 10.1038/s41598-020-63996-4 (PMC7184745; doi:10.1038/s41598-020-63996-4)
Supplement: Supplementary file 1 — Supplementary information. [file 41598_2020_63996_MOESM1_ESM.pdf]

New screening system using Twist1 promoter activity identifies dihydrorotenone as a potent drug targeting cancer-associated fibroblasts

Eunmyong Lee[1*], So-Young Yeo[2*], Keun-Woo Lee[2*], Jin A Lee[3], Kyeong Kyu Kim[2,3‡], Seok-Hyung Kim[,2,4‡]

Supplementary Methods

Histological analysis and immunohistochemistry

For immunostaining, slides were deparaffinized and antigen retrieval was performed in Tris-EDTA buffer (pH 9.25). After quenching peroxidase activity and blocking nonspecific binding, slides were incubated with anti-Twist1 (Cat: ab50887, Abcam, Cambridge, MA), anti-alpha-SMA (Cat: m0851, DAKO, Santa Clara, CA), anti-Ki-67 (Cat: 8D5, Cell Signaling, Danvers, MA), and anti-cleaved caspase-3 antibody (Cat: D175, Cell Signaling, Danvers, MA) for 2 hours. Slides were developed with AEC (3-amino-9-ethylcarbazole) solution (Cat: sk-4205, Vector), counterstained with hematoxylin, dehydrated, and mounted with mounting media (Cat: 008010, Life technology).

Supplementary Table 1. Primer sequences used in this study

Primers	Sequence 5’ → 3’
Twist1 forward	GTCCGCAGTCTTACGAGGAG
Twist1 reverse	CCAGCTTGAGGGTCTGAATC
FSP1 forward	GATGAGCAACTTGGACAGCAA
FSP1 reverse	CTGGGCTGCTTATCTGGGAAG
FAP forward	TCAACTGTGATGGCAAGAGCA
FAP reverse	TAGGAAGTGGGTCATGTGGGT
PDGFRα forward	AACCGTGTATAAGTCAGGGGA
PDGFRα reverse	GCATTGTGATGCCTTTGCCTT
PDGFRβ forward	TGATGCCGAGGAACTATTCATCT
PDGFRβ reverse	TTTCTTCTCGTGCAGTGTCAC
α-SMA forward	AGGGGGTGATGGTGGGAATG
α-SMA reverse	GCCCATCAGGCAACTCGTAAC
IL1-β forward	CTCGCCAGTGAAATGATGGCT
IL1-β reverse	GTCGGAGATTCGTAGCTGGCT
IL-6 forward	AACCTGAACCTTCCAAAGATGG
IL-6 reverse	TCTGGCTTGTTCCTCACTACT
IL-8 forward	GAATCCTCAGCCCTCTTCAAAAAC
IL-8 reverse	GCCAAGGAGTCGTAAAGAACTTAG
CXCL1 forward	CATGCCAGCCACTGTGATAGA
CXCL1 reverse	ATTCCCCTGCCTTCACAATG
CXCL2 forward	GGTTTGCAGATATTCTCTAGTCATTTGT
CXCL2 reverse	GGATTCCTCAGCCTCTATCACAGT
18S rRNA forward	GTAACCCGTTGAACCCCATT
18S rRNA reverse	CCATCCAATCGGTAGTAGCG
GAPDH forward	GAAGATGGTGATGGGATTTCCA
GAPDH reverse	GATTCCACCCATGGCAAATT

Related to Figure 2a

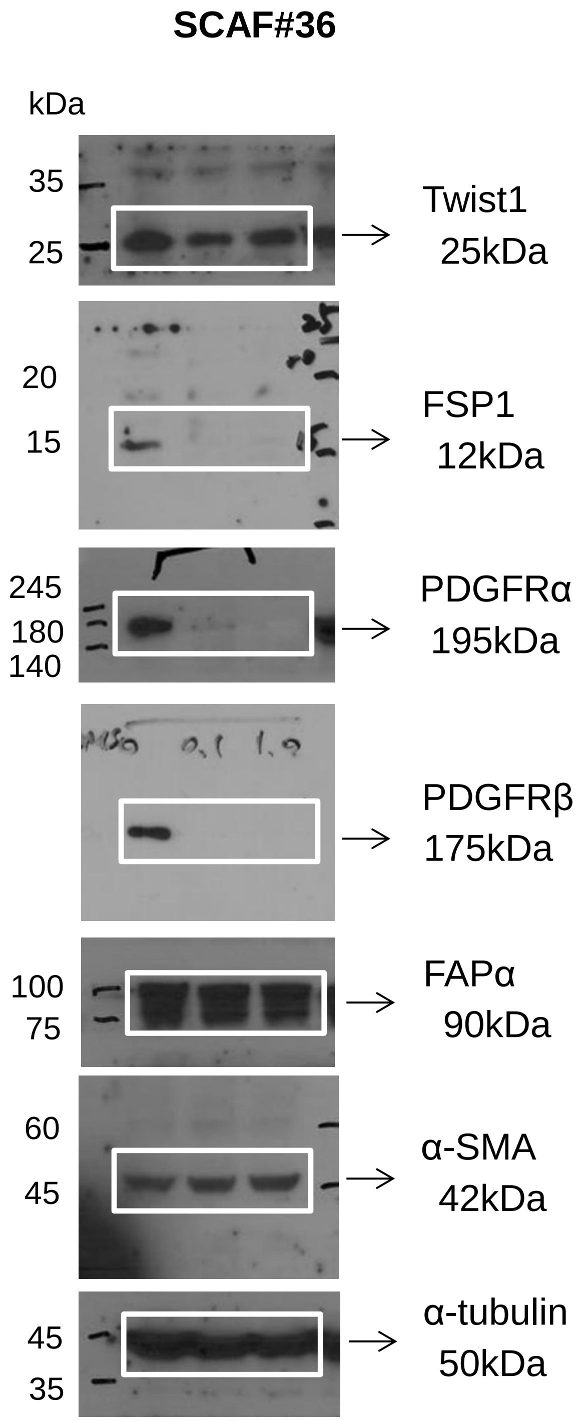

Supplementary Figure 2

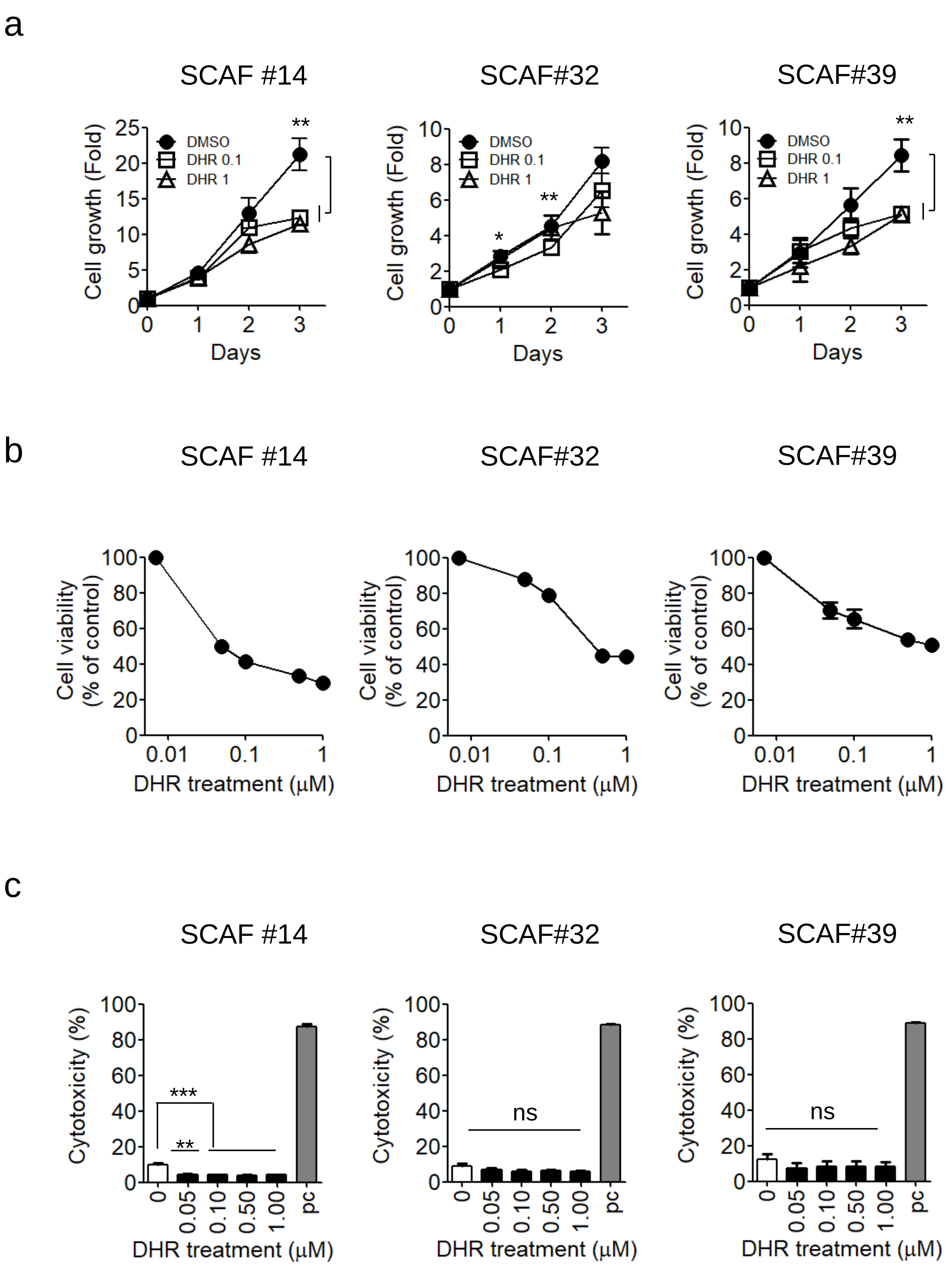

a

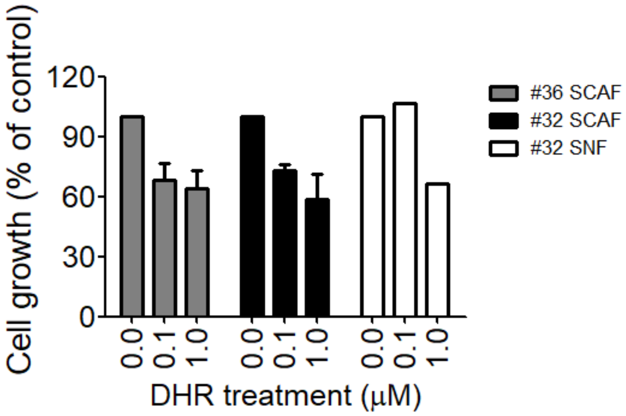
Cell growth (% of control)
120
90
60
30
0
0.0
0.1
1.0
0.0
0.1
1.0
0.0
0.1
1.0
DHR treatment (µM)
#36 SCAF
#32 SCAF
#32 SNF

b

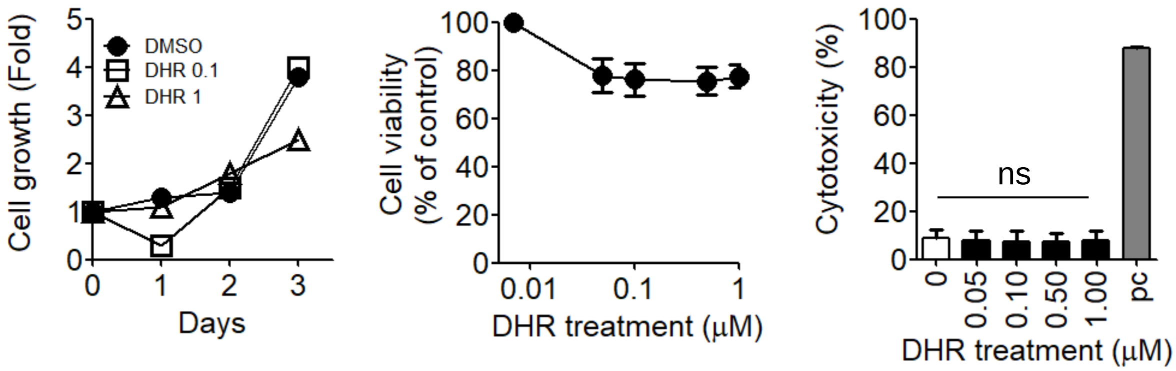
Cell growth (Fold)
5
4
3
2
1
0
DMSO
DHR 0.1
DHR 1
0
1
2
3
Days
Cell viability (% of control)
100
80
60
40
20
0
0.01
0.1
1
DHR treatment (µM)
Cytotoxicity (%)
100
80
60
40
20
0
ns
0
0.05
0.10
0.50
1.00
pc
DHR treatment (µM)

MKN28

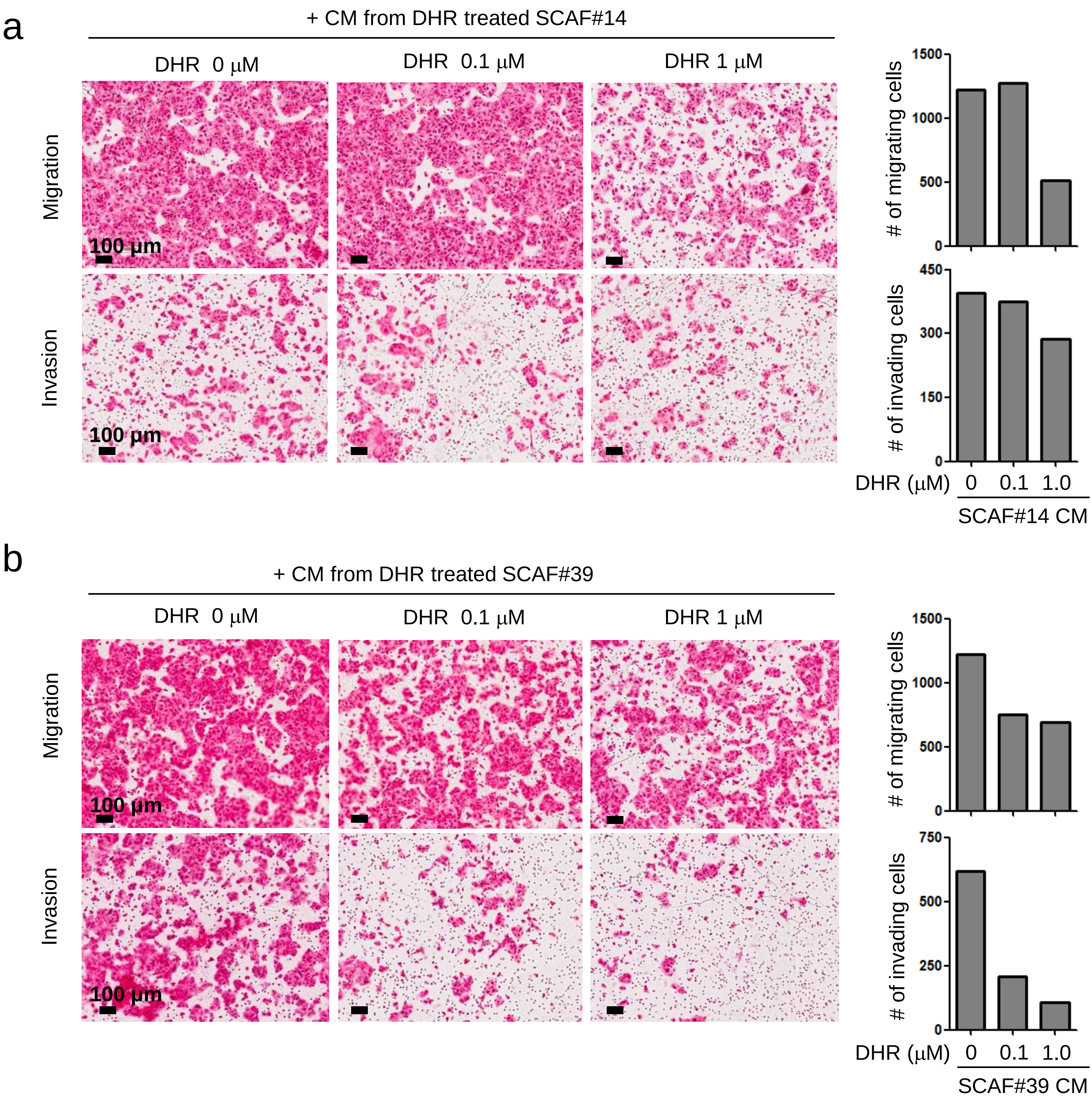
a
+ CM from DHR treated SCAF#14
DHR 0 µM
DHR 0.1 µM
DHR 1 µM
Migration
Invasion
100 µm
100 µm
# of migrating cells
1500
1000
500
0
# of invading cells
450
300
150
0
DHR (µM) 0 0.1 1.0
SCAF#14 CM
b
+ CM from DHR treated SCAF#39
DHR 0 µM
DHR 0.1 µM
DHR 1 µM
Migration
Invasion
100 µm
100 µm
# of migrating cells
1500
1000
500
0
# of invading cells
750
500
250
0
DHR (µM) 0 0.1 1.0
SCAF#39 CM

SNU668 Supplementary Figure 5

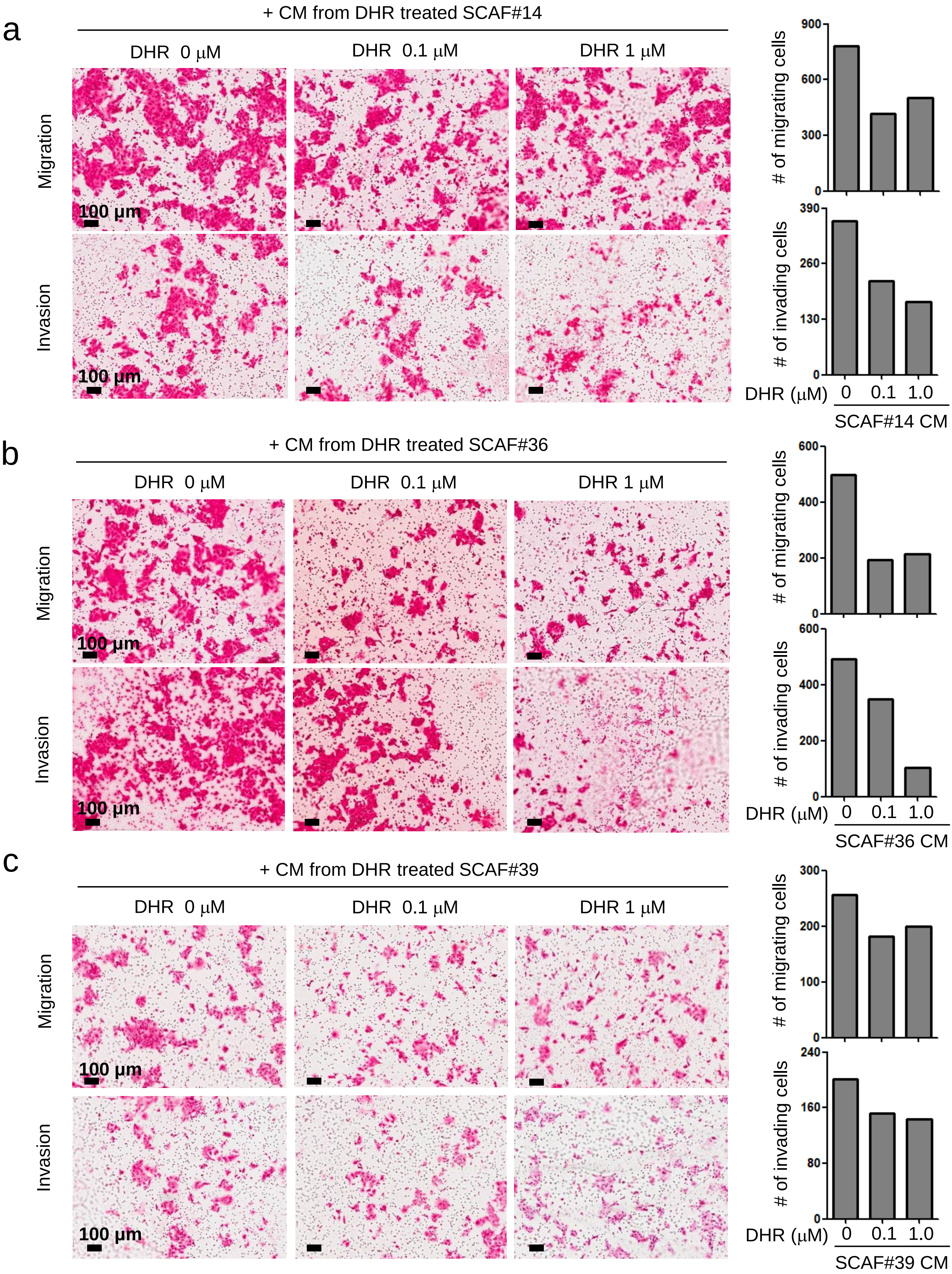

Supplementary Figure 6

MKN28

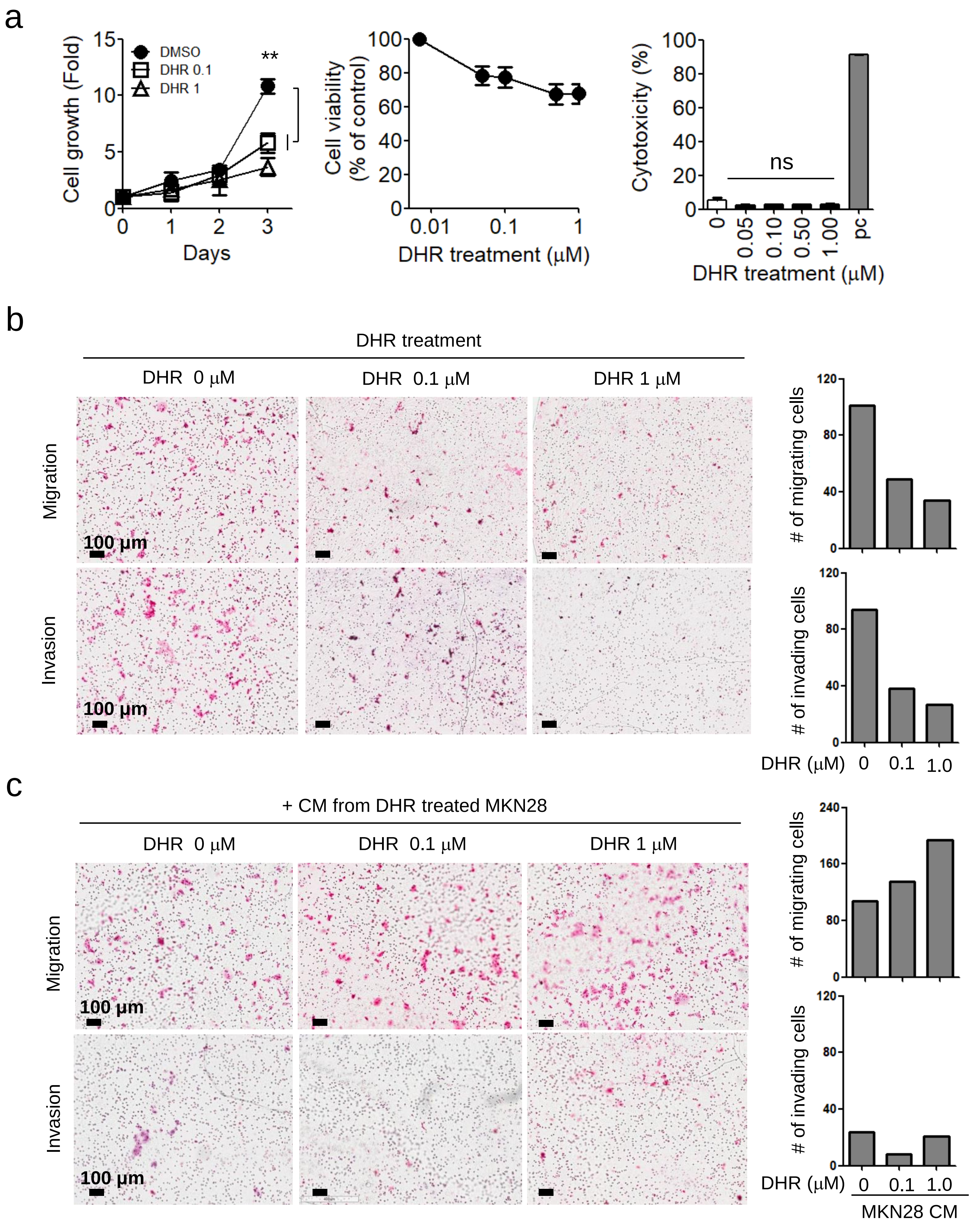

Supplementary Figure 7

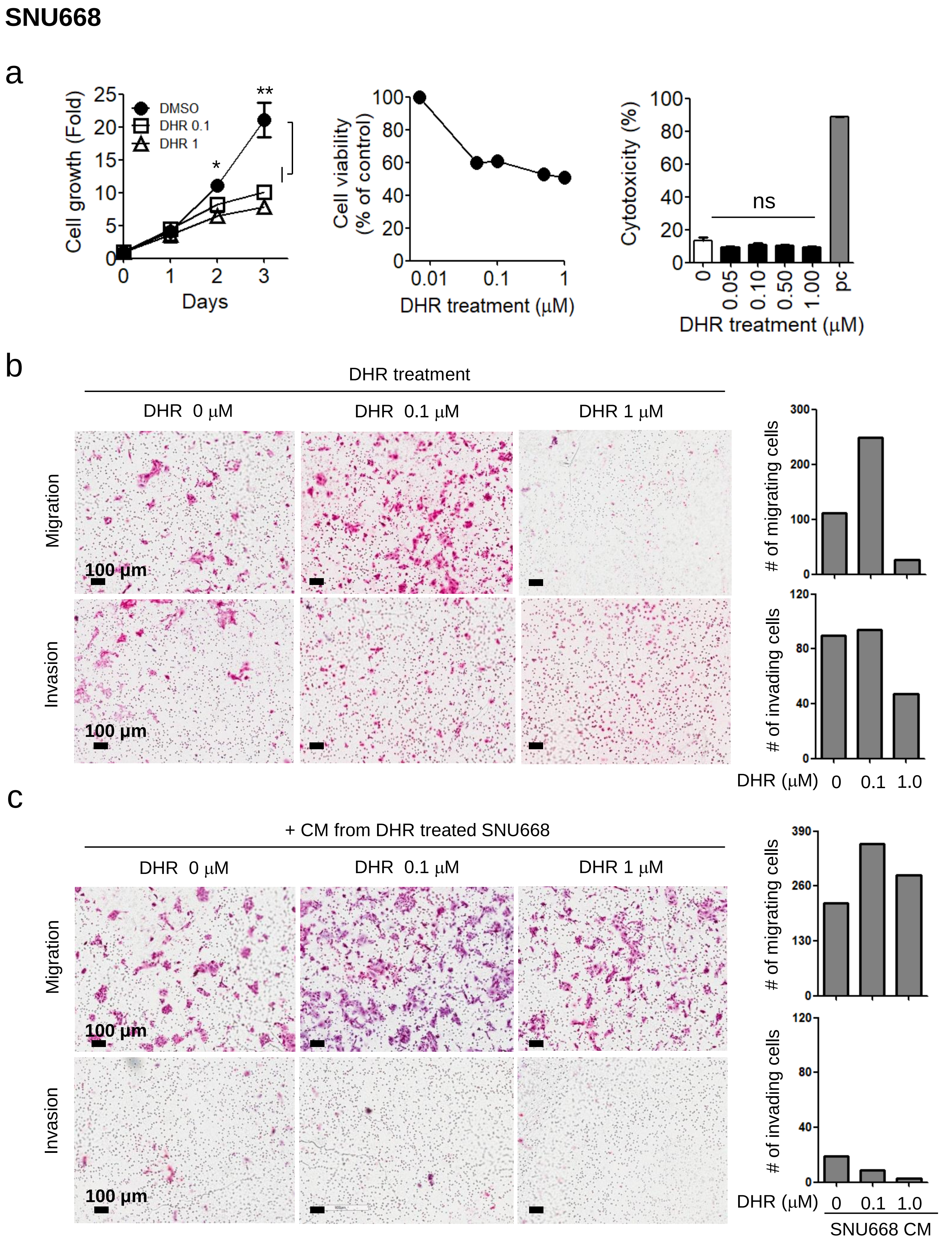

Supplementary Figure 8

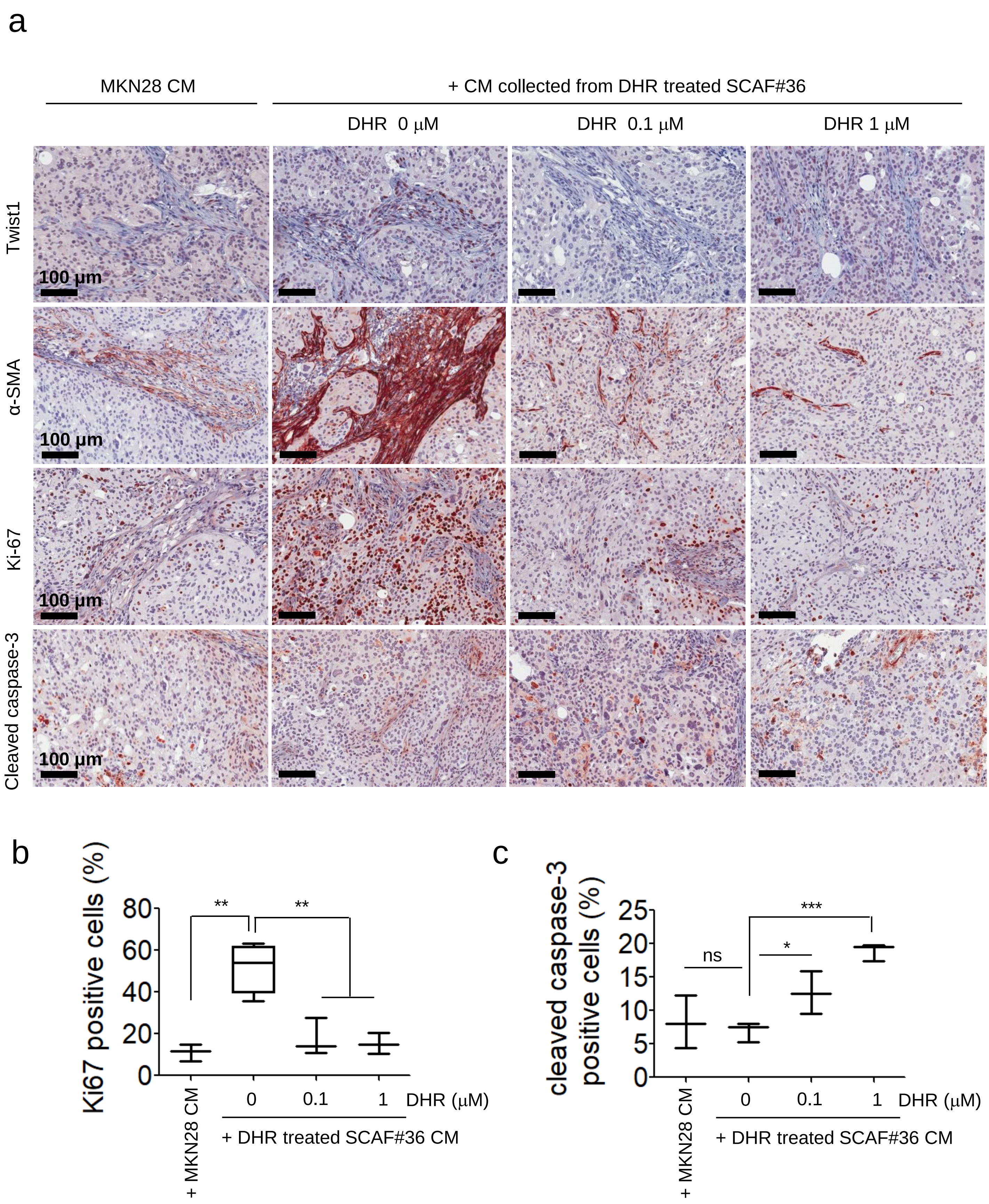

Figure Legend

Supplementary Figure 1 Uncropped images of western blots shown in Figure 2a with size markers are presented.

Supplementary Figure 2. DHR treatment decreases growth rate of CAFs without inducing significant direct cytotoxicity in CAFs SCAF#14, SCAF#32 and SCAF#39 cell proliferation, viability, and cytotoxicity under three days of DHR treatment. **(a)** SCAF#14 (**left panel**), SCAF#32 (**middle panel**), and SCAF#39 (**right panel**) cells were treated with 0.1 μM or 1 μM DHR, and cell proliferation was counted daily. Cell growth was normalized to day 0. Symbols represent the means ± SEM of more than four independent experiments. Differences were evaluated by two-tailed student's t-test. * $P < 0.05$; ** $P < 0.01$. **(b)** After 72 hours of DHR treatment, cell viability of SCAF#14 (**left panel**), SCAF#32 (**middle panel**), and SCAF#39 (**right panel**) cells was assessed using an EZ-cytox kit. Symbols represent the means ± SEM of more than three independent experiments. **(c)** The supernatants from SCAF#14 (**left panel)**, SCAF#32 (**middle panel**), and SCAF#39 (**right panel**) cells treated with DHR for 72 hours were assessed for LDH release with an EZ-LDH cytotoxicity assay kit. Experiments were done in triplicate. Bars represent the means ± SEM of more than three independent experiments. Differences were evaluated by two-tailed student's t-test.

Supplementary Figure 3 NFs are less sensitive to DHR treatment (a) SCAF#36, SCAF#32 and SNF#32 cell growth under three days of 0.1 μM or 1 μM DHR treatment. **(b)** SNF#32 cell proliferation (**left panel)**, viability (**middle panel)**, and cytotoxicity (**right panel)** under three days of 0.1 μM or 1 μM DHR treatment. Experiments were done in triplicate. Symbols and bars represent the means ± SEM. Differences were evaluated by two-tailed student's t-test.

Supplementary Figure 4. DHR treatment suppresses the tumor-promoting ability of CAFs MKN28 human stomach cancer cells were incubated with appropriate conditioned medium for two days and analyzed to assess their capacity for migration and invasion. Conditioned media were prepared from SCAF#14 cells or SCAF#39 cells treated with DMSO or DHR for three days. Migration and invasion of MKN28 cells treated with conditioned medium from SCAF#14 cells **(a)** and SCAF#39 cells **(b)**.

Supplementary Figure 5. DHR treatment suppresses the tumor-promoting ability of CAFs SNU668 human stomach cancer cells were incubated with appropriate conditioned medium for two days and analyzed to assess their capacity for migration and invasion. Conditioned media were prepared from SCAF#14, SCAF#36, or SCAF#39 cells treated with DMSO or DHR for three days. Migration and invasion of MKN28 cells treated with conditioned medium from SCAF#14 cells **(a),** SCAF#36 cells **(b)** and SCAF#39 cells **(c)**.

Supplementary Figure 6 Direct DHR treatment inhibits proliferation, migration and invasion of MKN28 cells. (a) Proliferation, viability, and cytotoxicity of MKN28 human stomach cancer cells under three days of DHR treatment. **(Left panel)** MKN28 cells were treated with 0.1 μM or 1 μM DHR, and cell proliferation was counted daily. Cell growth was normalized to day 0. Symbols represent the means ± SEM of three independent experiments. Differences were evaluated by two-tailed student's t-test. ** $P < 0.01$. **(Middle panel)** After 72 hours of DHR treatment, cell viability of MKN28 cells was assessed using an EZ-cytox kit. Symbols represent the means ± SEM of three independent experiments. **(Right panel)** The supernatants from MKN28 cells treated with DHR for 72 hours were assessed for LDH release with an EZ-LDH cytotoxicity assay kit. Experiments were done in triplicate. Bars represent the means ± SEM of three independent experiments. Differences were evaluated by two-tailed student's t-test. **(b)** MKN28 cells were treated with DMSO or DHR for three days and analyzed to assess their capacity for migration and invasion. **(c)** MKN28 human stomach cancer cells were incubated with appropriate conditioned medium for two days and analyzed to assess their capacity for migration and invasion. Conditioned media were prepared from MKN28 cells treated with DMSO or DHR for three days.

Supplementary Figure 7 Direct DHR treatment inhibits proliferation, migration and invasion of SMU668 cells.(a) Proliferation, viability, and cytotoxicity of SNNU668 human stomach cancer cells under three days of DHR treatment. **(Left panel)** SNU668 cells were treated with 0.1 μM or 1 μM DHR, and cell proliferation was counted daily. Cell growth was normalized to day 0. Symbols represent the means ± SEM of four independent experiments. Differences were evaluated by two-tailed student's t-test. * $P < 0.05$ (DMSO versus DHR 1 μM treatment, day2); ** $P < 0.01$. **(Middle panel)** After 72 hours of DHR treatment, cell viability of SNU668 cells was assessed using an EZ-cytox kit. Symbols represent the means ± SEM of three independent experiments. **(Right panel)** The supernatants from SNU668 cells treated with DHR for 72 hours were assessed for LDH release with an EZ-LDH cytotoxicity assay kit. Experiments were done in triplicate. Bars represent the means ± SEM of three independent experiments. Differences were evaluated by two-tailed student's t-test. **(b)** SNU668 cells were treated with DMSO or DHR for three days and analyzed to assess their capacity for migration and invasion. **(c)** SNU668 human stomach cancer cells were incubated with appropriate conditioned medium for two days and analyzed to assess their capacity for migration and invasion. Conditioned media were prepared from SNU668 cells treated with DMSO or DHR for three days.

Supplementary Figure 8 Tumors subjected to DHR-treated SCAF#36 conditioned medium shows less proliferation and more cell death (a) Representative photomicrography of Twist1, alpha-SMA, Ki-67 and cleaved caspase-3 stained microscopic sections of corresponding tumors. Scale bar, 100 µm. **(b-c)** The percentage of Ki-67 **(b)** or cleaved caspase-3 **(c)** positive cells per microscopic field was calculated from a total 3 fields per tumor. Whiskers represent mean with min to max. Difference was evaluated by two- tailed student's t-test. * $P < 0.05$; ** $P < 0.01$; *** $P < 0.001$.
